# Living Alone and Advance Care Planning: Do Neighborhood Cohesion and Disorder Matter?

**DOI:** 10.1093/geroni/igaf122.150

**Published:** 2025-12-31

**Authors:** Kedong Ding, Yifan Lou, Deborah Carr

**Affiliations:** Case Western Reserve University, Cleveland, Ohio, United States; Virginia Commonwealth University, Richmond, Virginia, United States; Boston University, Boston, Massachusetts, United States

## Abstract

More than16 million U.S. adults ages 65+ live alone. Given the centrality of coresidential family for older adults’ end-of-life preparation, it is unclear how those who live alone engage in advance care planning (ACP). Neighborhood characteristics like cohesion may enhance ACP, whereas disorder may impede it. Thus, our goal is to examine the association between living alone and three dimensions of ACP, and the extent to which these associations are moderated by self-reported neighborhood social cohesion (e.g., friendliness of people, availability of help when needed) and physical disorder (e.g., vandalism/graffiti, feeling safe alone after dark). We used data from 9,296 participants aged 65+ in the Health and Retirement Study (HRS) 2016 and 2018. ACP outcomes include living will, durable power of attorney for healthcare (DPAHC), and informal discussion. Multivariable analyses were adjusted family structure (marital status and number of children), sociodemographic, and health covariates. We find that living alone significantly increased the likelihood of having a living will, DPAHC, and discussion by 38%, 36%, and 30%, respectively. Living in neighborhoods with the highest cohesion (top 25-percentile) and lowest disorder (bottom 25-percentile) significantly increased the likelihood of formal ACP (i.e., living will and DPAHC), but not for informal discussions. Additionally, living in a socially cohesive neighborhood significantly increased the positive effect of living alone on having DPAHC (OR = 1.42). Gender-specific analyses showed that these moderating effects were particularly pronounced among women. We discussed the policy and practice implications of the role of neighborhood environment in living alone and ACP.

